# The safety and prognosis of radical surgery in colorectal cancer patients over 80 years old

**DOI:** 10.1186/s12893-023-01938-3

**Published:** 2023-02-28

**Authors:** Fu-Qiang Zhao, Yu-Juan Jiang, Wei Xing, Wei Pei, Jian-Wei Liang

**Affiliations:** 1 Department of Colorectal Surgery, National Cancer Center/National Clinical Research Center for Cancer/Cancer Hospital, Chinese Academy of Medical Sciences and Peking Union Medical College, Beijing, 100021 China; 2 Department of General Surgery, Hebei Province Hospital of Chinese Medicine; Affiliated Hospital of Hebei University of Chinese Medicine, Shijiazhuang, 050013 Hebei China; 3 Department of Minimally Invasive Surgery, Beijing Chest Hospital, Capital Medical University; Beijing Tuberculosis and Thoracic Tumor Research Institute, Beijing, China

**Keywords:** Elderly, Colorectal cancer, Surgery, Safety, Survival

## Abstract

**Objective:**

The purpose of this study was to assess the safety and feasibility of radical surgery and to investigate prognostic factors influencing in colorectal cancer (CRC) patients over the age of 80.

**Methods:**

Between January 2010 and December 2020, 372 elderly CRC patients who underwent curative resection at the National Cancer Center were enrolled in the study. Preoperative clinical characteristics, perioperative outcomes, and postoperative pathological features were all collected.

**Results:**

A total of 372 elderly patients with colorectal cancer were included in the study, including 226 (60.8%) men and 146 (39.2%) women. A total of 219 (58.9%) patients had a BMI < 24 kg/m^2^, and 153 (41.1%) patients had a BMI ≥ 24 kg/m^2^. The mean operation time and intraoperative blood loss were 152.3 ± 58.1 min and 67.6 ± 35.4 ml, respectively. The incidence of overall postoperative complications was 28.2% (105/372), and the incidence of grade 3–4 complications was 14.7% (55/372). In the multivariable Cox regression analysis, BMI ≥ 24 kg/m^2^ (HR, 2.30, 95% CI, 1.27–4.17; *P* = 0.006) and N1-N2 stage (HR: 2.97; 95% CI, 1.48–5.97; *P* = 0.002) correlated with worse CSS.

**Conclusion:**

The findings of this study showed that radical resection for CRC is safe and feasible for patients over the age of 80. After radical resection, BMI and N stage were independent prognostic factors for elderly CRC patients.

## Introduction

Colorectal cancer (CRC) is one of the most common causes of cancer death worldwide, and its morbidity and mortality are on the rise [1, 2]. With the expansion of the population and the improvement of living standards, the ageing of the population continues to increase [3]. Therefore, in clinical practice, the proportion of older patients receiving surgical treatment for colorectal cancer is increasing. Elderly patients with CRC have unusual clinicopathological features and genetic backgrounds [4, 5]. In addition, these individuals often have comorbidities such as cardiovascular and cerebrovascular diseases and diabetes and often need more rigorous and prudent standardized management during the perioperative period [6]. According to the clinical consensus and guidelines, adjuvant treatment such as chemotherapy and radiotherapy is not recommended for CRC patients older than 80 years of age regardless of TNM stage, but traditional prognostic indicators may not be suitable for elderly patients with CRC over 80 years old [8–15]. Therefore, the main purpose of the present study was to demonstrate the safety and feasibility of radical surgery for CRC in elderly patients over 80 years of age, to evaluate the prognosis of elderly CRC patients without adjuvant therapy using the tumour-specific survival rate, and to comprehensively explore relevant prognostic factors.

## Materials and methods

### Patients

From January 2010 to December 2020, all consecutive CRC patients older than 80 years of age who underwent curative resection at the National Cancer Center/National Clinical Research Center for Cancer/Cancer Hospital, Chinese Academy of Medical Sciences and Peking Union Medical College, were retrospectively collected and analysed. The inclusion criteria were as follows: (1) age 80 or above; (2) pathologically confirmed colorectal adenocarcinoma; (3) no evidence of distant metastasis; and (4) no adjuvant therapy, such as radiotherapy or chemotherapy, after the operation. Patients who underwent emergency surgery or had other malignant tumours were excluded from the analysis. The study was approved by the Ethics Committee of the Cancer Hospital, Chinese Academy of Medical Sciences and was conducted in accordance with the Declaration of Helsinki and Ethical Guidelines for Clinical Research. All patients provided written informed consent.

Clinical characteristics, perioperative variables, pathological results and survival outcomes for all patients were obtained from the medical records, operation records, and pathology records in our hospital database. Postoperative complications were assessed using the Clavien-Dindo classification (CD) categories and were defined as any condition that occured within 30 days after surgery that affected the normal recovery process and required conservative or surgical intervention [16]. All procedures were performed by surgeons with more than 20 years of experience in colorectal surgery. The American Joint Committee on Cancer (AJCC, eighth edition) staging system was used for tumour staging.

### Surgical procedures

Curative-intent surgery was performed for all patients diagnosed with CRC. All patients were placed in the modified lithotomy position, and patients underwent laparoscopic surgery or open surgery. In principle, laparoscopic surgery was performed by the five-port method under general anaesthesia. The TME and CME techniques were standardized as described previously. Briefly, the concept of TME or CME was based upon continuous sharp separation of the visceral fascial layer from the parietal layer. Then, the entire mesentery, completely covered by the visceral fascial layer, was obtained, ensuring safe exposure and ligation of the beginning of the supplying artery. The extent of surgery was determined by the location of the tumour and the pattern of underlying lymphatic metastases.

#### Follow-up

The long-term outcome of the present study was the 3-year cancer-specific survival (CSS) rate. All patients received a follow-up survey every 2 months for the first 2 years and every 6 months for the next 3 years. The postoperative review examinations included physical examination, biomarkers (CEA and CA-199); CT scans of the chest, abdomen, and pelvis, and colonoscopy if necessary. CSS was defined as the time between the date of surgery and the date of death from cancer. Disease free survival (DFS) is defined as the time from surgery to disease recurrence or last follow-up. Overall survival (OS) is defined as the time from surgery to the time of death of the patient for any cause or the time to the last follow-up. The deadline for follow-up in this study was December 2022.

#### Statistical analysis

The mean ± standard deviation was used to represent quantitative data, while frequencies and percentages were used to represent categorical variables. The factors predicting CSS were identified using univariate and multivariate Cox regression models. To analyse the 3-year CSS of the patients in different groups, the Kaplan–Meier survival method was used, and significant differences in CSS were compared using the log-rank test. The variables that were statistically significant (*P* < 0.20) in univariate analysis were then tested in multivariate analysis using a Cox regression model, and the effect of each variable was assessed using the hazard ratio (HR) and 95% confidence interval (95% CI). *P* values less than 0.05 were considered statistically significant. IBM SPSS Statistics software version 24.0 was used for statistical analyses (IBM Corporation, Armonk, NY, USA).

## Results

### Short-term outcomes

Table 1 summarizes the baseline characteristics of the patients. Among the 372 elderly CRC patients included in this study, 226 (60.8%) were male, and 146 (39.2%) were female. Among all patients, 36 (9.7%) patients were older than 85 years. In addition, 104 (27.9%) patients had tumours in the right colon, 132 (35.5%) patients had tumours in the left colon, and 136 (36.6%) patients had tumours in rectum. The perioperative outcomes and pathological results are listed in Table 2. The mean operation time and intraoperative blood loss were 152.3 ± 58.1 min and 67.6 ± 35.4 ml, respectively. Regarding postoperative recovery, the mean postoperative hospital stay was 11.0 ± 5.6 days, and only one (0.2%) patient died in the perioperative period.

**Table 1:** Baseline characteristics

Variables	N = 372
Gender	
Male	226 (60.8)
Female	146 (39.2)
Age at operation (years old)	
< 85	336 (90.3)
≥ 85	36 (9.7)
Body mass index (kg/m^2^)	
< 24	219 (58.9)
≥ 24	153 (41.1)
ASA score	
II	218 (58.6)
III	154 (41.4)
Preoperative albumin (g/L)	
< 35	74 (19.9)
≥ 35	298 (80.1)
Preoperative HGB (g/L)	
< 110	77 (20.7)
≥ 110	295 (79.3)
Habits	
Drinking	72 (19.4)
Smoking	90 (24.2)
Comorbidity	
Hypertension	170 (45.7)
Diabetes mellitus	48 (12.9)
Coronary artery disease	36 (9.7)
Arrhythmia	70 (18.8)
Respiratory diseases	46 (12.4)
Other	22 (5.9)
Previous abdominal surgery	76 (20.4)
Tumour location	
Right colon	104 (30.0)
Left colon	132 (35.5)
Rectum	136 (36.6)

**Table 2:** Pathological data and perioperative outcome

Variables	N = 372
T stage	
T1-T2	76 (20.4)
T3-T4	296 (79.6)
N stage	
N0	204 (54.8)
N1-N2	168 (45.2)
Tumour grade	
I	86 (23.1)
II	195 (52.4)
III	91 (24.5)
Tumour size (cm, mean ± SD)	4.7 ± 2.2
Perineural invasion	106 (28.5)
Lymphatic invasion	110 (29.6)
LN harvest (days, mean ± SD)	17.7 ± 8.4
Surgical procedure	
Open	164 (44.1)
Laparoscope	208 (55.9)
Operative time (min, mean ± SD)	152.3 ± 58.1
Estimated intraoperative blood loss (ml, mean ± SD)	67.6 ± 35.4
Postoperative complications	105 (28.2)
Grade 3–4 postoperative complications	55 (14.7)
Time to first flatus (days, mean ± SD)	5.1 ± 2.2
Postoperative hospital stay (days, mean ± SD)	11.0 ± 5.6
Re-operation	15 (4.0)
Mortality	1 (0.2)

Table 3 lists the postoperative complications of the 372 elderly CRC patients. The incidence rates of overall complications, grade 1–2 complications, and grade 3–4 complications were 28.2%, 13.5%, and 14.7%, respectively. Among the overall complications, abdominal abscess (5.4%), anastomotic leakage (4.6%), and ileus (4.6%) were the most common. The most common grade 3–4 complication was urinary retention (2.4%), followed by pleural effusion (2.2%) and abdominal abscess (1.9%).

**Table 3:** Overall and grade 3–4 postoperative complications of 372 elderly patients

	Grade 1–2 complications		Grade 3–4 complications		All complications	
	n	%	n	%	n	%
Complications						
Total	50	13.5	55	14.7	105	28.2
Cardiac disorders						
Arrhythmia	5	1.3	6	1.6	11	2.9
Cardiac failure	3	0.8	1	0.2	4	1.0
Acute coronary sy ndrome	1	0.3	2	0.5	3	0.8
Hypertensive emergencies	1	0.3	0	0	1	0.3
Respiratory disorder						
Pneumonia	4	1.1	6	1.6	10	2.7
Pleural effusion	2	0.5	8	2.2	10	2.7
Atelectasis	3	0.8	5	1.3	8	2.1
Gastrointestinal haemorrhage						
Anastomotic leakage	11	3.0	6	1.6	17	4.6
Ileus	11	3.0	6	1.6	17	4.6
GastrointestinaI hemorrhage	4	1.1	2	0.5	6	1.6
Renal and urinary disorders						
Urinary infection	7	1.9	1	0.2	8	2.1
Renal failure	0	0	1	0.2	1	0.2
Urinary retention	3	0.8	9	2.4	12	3.2
Other disorders						
Abdominal abscess	13	3.5	7	1.9	20	5.4
Rectovaginal fistula	0	0	1	0.2	1	0.2
Intra-abdominal haemorrhage	3	0.8	4	1.1	7	1.9
Wound infection	12	3.2	2	0.5	14	3.7
Pulmonary embolism	0	0	2	0.5	2	0.5

### Survival analysis

**Fig. 1:**
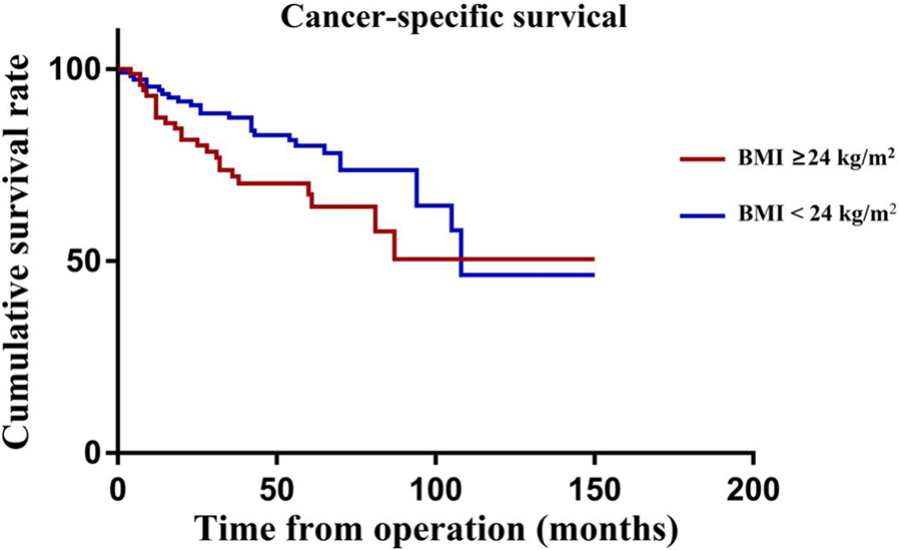
Cancer-specific survival curve of overweight group and control group

**Fig. 2:**
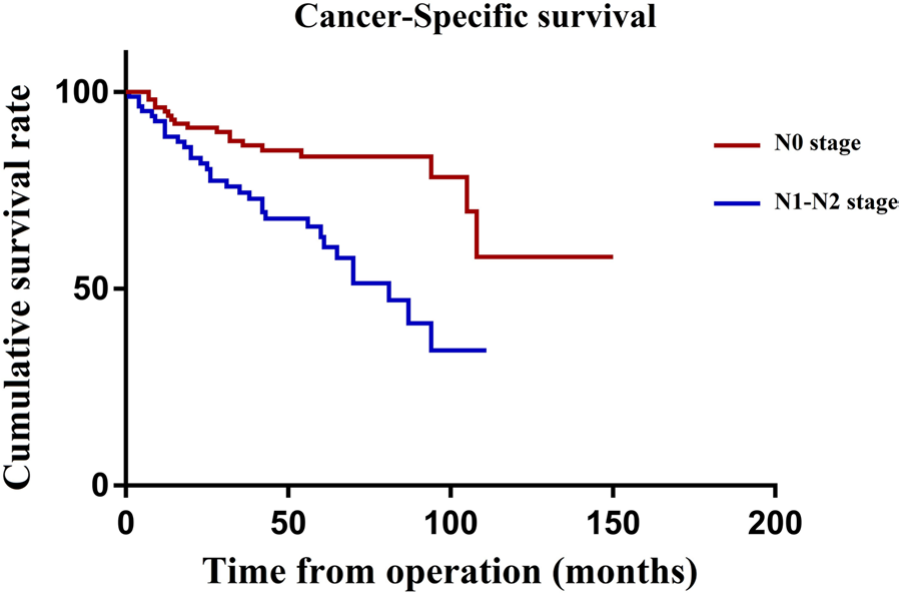
Cancer-specific survival curve of N0 group and N1-2 group

**Fig. 3:**
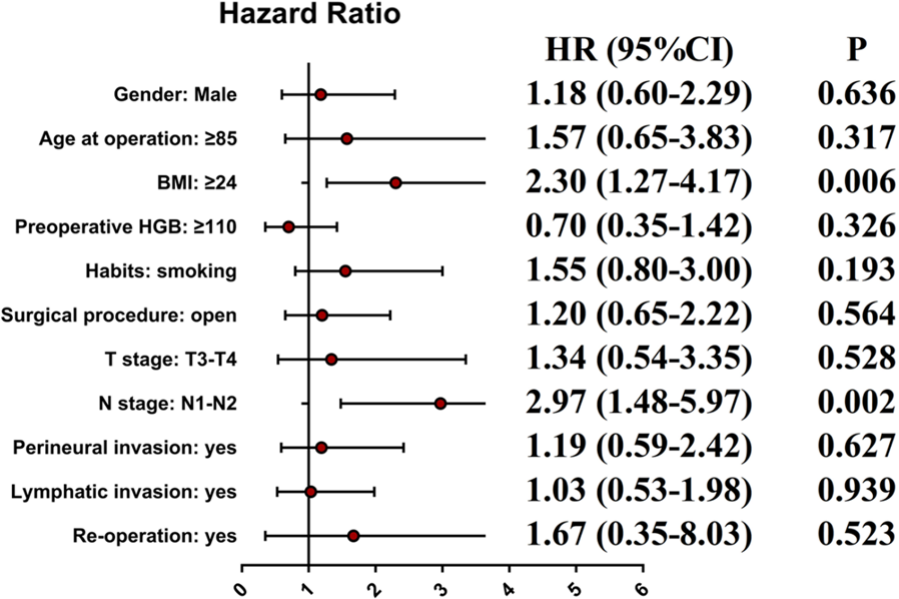
Forest plots for Cancer-specific survival of elderly CRC patients after curative resection based on multivariable COX proportional hazard model

The mean follow-up period for the whole group was 60 months (range, 29–150 months). During this period, 130 of the 372 patients died (34.9%). Among them, 102 died from tumour recurrence or metastasis (27.4%). In the univariate analysis, sex, age, BMI, preoperative HGB, lifestyle habits, surgical procedure, T stage, N stage, perineural invasion, lymphatic invasion, and reoperation significantly affected CSS (*P* < 0.2). These variables were thus incorporated into the multivariate analysis, and the results revealed that the CSS was significantly affected by BMI (HR, 2.30, 95% CI, 1.27–4.17; *P* = 0.006) and N stage (HR: 2.97; 95% CI, 1.48–5.97; *P* = 0.002) (Table 4). The Kaplan curves showed that the CSS rate of patients was affected by the BMI (*P* = 0.046, Fig. 1) and N stage (*P* < 0.001, Fig. 2). Figure 3 shows the forest plots for CSS of elderly CRC patients based on the multivariable Cox proportional hazard model. Next, we performed prognostic analysis on DFS and OS, and found that BMI and N stage were independent prognostic factors (Tables 5 and 6).

**Table 4:** Univariate and multivariate Cox regression analyses of cancer specific survival in 372 elderly patients after curative resection

Variables	Cancer specific survival			
	Univariate analysis		Multivariate analysis	
	HR(95%CI)	P	HR(95%CI)	P
Gender: male/female	1.63 (0.88–3.02)	0.118	1.18 (0.60–2.29)	0.636
Age at operation: ≥ 85/ < 85	1.84 (0.83–4.08)	0.137	1.57 (0.65–3.83)	0.317
ASA score: III/II	1.08 (0.62–1.90)	0.779		
Body mass index				
< 24	Reference	-	Reference	-
≥ 24	1.76 (1.01–3.05)	0.046	2.30 (1.27–4.17)	0.006
Preoperative albumin: ≥ 35/ < 35	0.74 (0.40–1.35)	0.322		
Preoperative HGB: ≥ 110/ < 110	0.63 (0.34–1.19)	0.158	0.70 (0.35–1.42)	0.326
Habits: drinking	1.46 (0.79–2.70)	0.233		
Habits: smoking	1.49 (0.83–2.69)	0.184	1.55 (0.80–3.00)	0.193
Comorbity: yes/no	0.78 (0.45–1.36)	0.382		
Previous abdominal surgery:yes/no	0.69 (0.33–1.48)	0.342		
Tumor location: left colon and rectum/ right colon	1.09 (0.58–2.04)	0.797		
Surgical procedure: open/laparoscope	1.46 (0.83–2.57)	0.185	1.20 (0.65–2.22)	0.564
Operative time: ≥ 135/ < 135	1.12 (0.65–1.94)	0.688		
T stage: T3-T4/T1-T2	2.28 (0.97–5.35)	0.058	1.34 (0.54–3.35)	0.528
N stage: N1-N2/N0	2.90 (1.63–5.18)	< 0.001	2.97 (1.48–5.97)	0.002
Tumor grade				
I	Reference	-		
II	0.71 (0.28–1.83)	0.483		
III	0.94 (0.33–2.67)	0.904		
Perineural invasion: yes/no	1.74 (0.95–3.19)	0.074	1.19 (0.59–2.42)	0.627
Lymphatic invasion	1.99 (1.14–3.47)	0.016	1.03 (0.53–1.98)	0.939
Grade 3–4 complications: yes/no	0.82 (0.35–1.92)	0.641		
Re-operation: yes/no	3.21 (0.77–13.37)	0.109	1.67 (0.35–8.03)	0.523

**Table 5:** Univariate and multivariate Cox regression analyses of overall survival in 372 elderly patients after curative resection

Variables	Overall survival			
	Univariate analysis		Multivariate analysis	
	HR(95%CI)	P	HR(95%CI)	P
Gender: male/female	1.51 (1.06–2.17)	0.024	1.31 (0.91–1.90)	0.151
Age at operation: ≥ 85/ < 85	1.65 (0.77–3.54)	0.199	0.56 (0.37–0.85)	0.107
ASA score: III/II	0.37 (0.25–0.54)	0.225		
Body mass index: ≥ 24/ < 24	1.99 (1.22–3.25)	0.006	1.45 (0.87–2.40)	0.005
Preoperative albumin: ≥ 35/ < 35	1.15 (0.80–1.66)	0.442		
Preoperative HGB: ≥ 110/ < 110	1.34 (0.83–2.16)	0.236		
Habits: drinking	0.93 (0.58–1.48)	0.754		
Habits: smoking	0.93 (0.58–1.48)	0.753		
Comorbity: yes/no	1.51 (1.03–2.20)	0.035	1.34 (0.90–1.99)	0.144
Previous abdominal surgery:yes/no	1.16 (0.76–1.79)	0.488		
Tumor location				
Left colon	Reference	-		
Right colon	0.94 (0.58–1.53)	0.805		
Rectum	1.00 (0.66–1.52)	0.997		
Surgical procedure: open/laparoscope	0.24 (0.16–0.35)	0.223	0.34 (0.23–0.53)	0.121
Operative time: ≥ 135/ < 135	1.07 (0.72–1.59)	0.734		
T stage: T3-T4/T1-T2	0.87 (0.57–1.32)	0.505		
N stage: N1-N2/N0	2.56 (1.72–3.80)	< 0.001	2.14 (1.40–3.26)	< 0.001
Tumor grade				
I	Reference	-		
II	2.48 (1.15–5.36)	0.021	1.66 (0.75–3.70)	0.215
III	3.34 (1.45–7.69)	0.005	1.71 (0.71–4.10)	0.230
Perineural invasion: yes/no	0.97 (0.67–1.39)	0.850		
Lymphatic invasion	0.95 (0.63–1.43)	0.812		
Grade 3–4 complications: yes/no	0.58 (0.36–0.92)	0.021	0.78 (0.48–1.27)	0.345
Re-operation: yes/no	1.59 (0.51–5.02)	0.427

**Table 6:** Univariate and multivariate Cox regression analyses of disease free survival in 372 elderly patients after curative resection

Variables	Disease free survival			
	Univariate analysis		Multivariate analysis	
	HR(95%CI)	P	HR(95%CI)	P
Gender: male/female	1.42 (1.01–1.99)	0.046	1.28 (0.90–1.82)	0.171
Age at operation: ≥ 85/ < 85	1.69 (0.82–3.45)	0.152	1.31 (0.62–2.75)	0.480
ASA score: III/II	1.01 (0.72–1.43)	0.939		
Body mass index: ≥ 24/ < 24	0.46 (0.32–0.66)	< 0.001	0.56 (0.37–0.85)	0.046
Preoperative albumin: ≥ 35/ < 35	1.85 (1.17–2.93)	0.008	1.38 (0.85–2.23)	0.190
Preoperative HGB: ≥ 110/ < 110	1.37 (0.87–2.14)	0.175	1.30 (0.81–2.10)	0.281
Habits: drinking	0.95 (0.61–1.47)	0.811		
Habits: smoking	1.11 (0.72–1.69)	0.640		
Comorbity: yes/no	1.27 (0.89–1.81)	0.188	1.17 (0.81–1.69)	0.397
Previous abdominal surgery: yes/no	1.24 (0.83–1.87)	0.293		
Tumor location				
Left colon	Reference	–		
Right colon	0.99 (0.63–1.57)	0.968		
Rectum	0.95 (0.64–1.42)	0.802		
Surgical procedure: open/laparoscope	0.96 (0.68–1.36)	0.813		
Operative time: ≥ 135/ < 135	1.20 (0.83–1.73)	0.338		
T stage: T3-T4/T1-T2	1.05 (0.69–1.60)	0.816		
N stage: N1-N2/N0	0.35 (0.24–0.50)	< 0.001	0.51 (0.34–0.76)	< 0.001
Tumor grade				
I	Reference	–		
II	2.36 (1.14–4.86)	0.020	1.85 (0.87–3.92)	0.109
III	3.38 (1.54–7.41)	0.002	2.26 (0.99–5.15)	0.053
Perineural invasion: yes/no	2.07 (1.41–3.03)	0.211		
Lymphatic invasion	1.07 (0.73–1.56)	0.729		
Grade 3–4 complications: yes/no	0.63 (0.41–0.98)	0.040	0.85 (0.53–1.35)	0.483
Re-operation: yes/no	1.24 (0.39–3.88)	0.718		

## Discussion

One of the biggest challenges in healthcare is the ageing population; in 2015, the life expectancy at birth was 82.9 years, with males expected to live to 80.5 years old and females expected to live to 85.1 years old. Elderly CRC patients are regarded as a special population with unique clinicopathological characteristics, and the increase in comorbidities typically observed in this population tends to increase the potential risks during the perioperative period. In the present study, we aimed to investigate the short-term safety and long-term prognosis of radical surgery for CRC in older adults over 80 years of age.

The safety of radical surgery for elderly patients with colorectal cancer is a concern for surgeons. Prior works have reported that the incidence of overall complications in elderly patients with CRC after radical surgery is 9.9–25.4%, and the incidence of grade 3–5 complications is 6.5–20.1% [12–15]. Our study showed that the incidence rates of overall complications, grade 1–2 complications, and grade 3–4 complications were 28.2%, 13.5%, and 14.7%, respectively, which were consistent with previous reports in the literature. In addition, this study revealed that the most common overall complication after radical resection of elderly patients with CRC is an abdominal abscess (5.4%), and the most common grade 3–4 postoperative complication is urinary retention (2.4%). Before surgery, we should pay attention to and try to improve the patient's general condition, perform transfusion, supplement albumin, carry out enteral nutrition to improve the patient's nutritional status, and actively treat basic diseases such as hypertension, heart disease, and diabetes. According to the blood supply and tension of the patient's intestinal tube, the operation was performed gently, and the principle of being sterile and tumour-free was strictly followed. Postoperative nutritional support should also be actively carried out to provide sufficient raw materials for the growth of the anastomotic mouth.

Along with the increase in material wealth, the incidence of obesity has increased and become a medical and social problem. Obesity is clearly associated with the incidence of CRC [17–22], and the relationship between obesity and colorectal cancer has been previously reported but remains controversial. Several studies have reported that a high BMI is associated with a poor prognosis in patients with CRC [20, 23], while other studies have reported that a high BMI is not related to prognosis [24, 25] or is even related to a better prognosis [26, 27]. This study explores the prognostic factors related to elderly patients with CRC after curative resection, and the results show that BMI ≥ 24 kg/m^2^ (HR, 2.30, 95% CI, 1.27–4.17; *P* = 0.006) and N1-N2 stage (HR: 2.97; 95% CI, 1.48–5.97; *P* = 0.002) were independent prognostic factors affecting CSS. Scarpa et al. grouped 595 CRC patients based on BMI and conducted postoperative follow-ups. Multivariate analysis showed that BMI > 30 kg/m^2^ was an independent risk factor for prognosis and recurrence after surgery (HR: 2.2; 95% CI, 1.3–3.9; *P* = 0.003) [28]. Doria-Rose et al. obtained similar results: patients with a high BMI, especially a BMI > 35 kg/m^2^, had a higher recurrence rate and poorer overall survival than those with a normal BMI [29]. The results of the above studies were basically consistent with our findings.

Over the past two decades, laparoscopic colorectal resection has grown in popularity. Laparoscopic colectomy is linked to better immunological and inflammatory responses, shorter hospitalization, and similar long-term oncologic outcomes compared to open surgery, according to a number of randomized, prospective clinical trials [30]. Nevertheless, the complexity of the pelvis' anatomical structure, the need for higher technical expertise during total mesorectal excision (TME), and the fact that colectomy preserves the autonomic nerves make minimally invasive surgery for rectal cancer contentious. Laparoscopic rectal cancer surgery has been shown to be safe and to result in better functional recovery and oncological outcomes than open surgery in a number of randomized controlled trials (RCTs) and meta-analyses [31]. Several recent studies have shown that laparoscopic rectal cancer resection might safely be performed irrespective of age [30, 32]. However, there is a lack of data about the long-term results of laparoscopic versus open resection in senior rectal cancer patients.

Particular attention is required when planning chemotherapy for elderly cancer patients because of reductions in organ function and pre-existing comorbidities. Most of the current randomized trials did not include many elderly patients. In 2012, Sanoff et al. [33] reported a cohort study combining four large databases of patients diagnosed with stage III CRC between 2004 and 2007. A total of 5489 patients with stage III CRC aged ≥ 75 years were analysed using covariate-adjusted and propensity score-matched proportional hazards models. Compared with surgery alone, 5-FU-based adjuvant chemotherapy had a significant survival benefit, whereas the addition of oxaliplatin to 5-FU-based chemotherapy provided no significant benefit over 5-FU alone, although it tended to improve prognosis. In future studies, we will use our data to further explore the efficacy of adjuvant therapy in older adults.

The most significant limitation of the present study is its retrospective nature, and only 372 patients were included, which may have caused some inherent selection bias. In addition, compared to rectal cancer, colon cancer is more likely to cause systemic consumption and lower BMI, and we did not calculate colon and rectal cancer separately. Therefore, multicentre, large-scale, prospective studies are warranted to verify our results.

In conclusion, our findings show that radical resection for CRC is safe and feasible for patients over the age of 80. After radical resection, BMI and N stage were independent prognostic factors for elderly CRC patients.

## Data Availability

The data that support the findings of this study are available on request from the corresponding author. The data are not publicly available due to privacy or ethical restrictions.

## Abbreviations

CSS: Cancer-specific survival
CRC: Colorectal cancer
BMI: Body mass index
CD: Clavien–Dindo classification
DFS: Disease free survival
OS: Overall survival
