# Correction to: The safety and prognosis of radical surgery in colorectal cancer patients over 80 years old

**DOI:** 10.1186/s12893-023-01965-0

**Published:** 2023-03-29

**Authors:** Fu-Qiang Zhao, Yu-Juan Jiang, Wei Xing, Wei Pei, Jian-Wei Liang

**Affiliations:** 1 Department of Colorectal Surgery, National Cancer Center/National Clinical Research Center for Cancer/Cancer Hospital, Chinese Academy of Medical Sciences and Peking Union Medical College, Beijing, 100021 China; 2 Department of General Surgery, Hebei Province Hospital of Chinese Medicine, Affiliated Hospital of Hebei University of Chinese Medicine, Shijiazhuang, China

Following publication of the original article [1], in this article the affiliation details for all the authors were incorrectly given and it should has shown below:

Fu-Qiang Zhao^1^, Yu-Juan Jiang^1^, Wei Xing^2*^, Wei Pei^1^, Jian-Wei Liang^1^

^1^Department of Colorectal Surgery, National Cancer Center/National Clinical Research Center for Cancer/Cancer Hospital, Chinese Academy of Medical Sciences and Peking Union Medical College, Beijing 100021, China

^2^Department of General Surgery, Hebei Province Hospital of Chinese Medicine; Affiliated Hospital of Hebei University of Chinese Medicine, Shijiazhuang, China

The original article has been corrected.
